# A label masked autoencoder for image-guided segmentation label completion

**DOI:** 10.1016/j.patter.2025.101455

**Published:** 2025-12-22

**Authors:** Jiaru Jia, Mingzhe Liu, Dongfen Li, Xin Chen, Ruili Wang, Linlin Zhuo, Keqin Li

**Affiliations:** 1 School of Data Science and Artificial Intelligence, Wenzhou University of Technology, Wenzhou 325035, China; 2 College of Biomedical Engineering, Wenzhou Medical University, Wenzhou 325000, China; 3 College of Computer Science and Cyber Security, Chengdu University of Technology, Chengdu 610059, China; 4 Massey University, Auckland 2820, New Zealand; 5 Department of Computer Science, State University of New York, New York, NY 12561, USA

**Keywords:** semantic segmentation, transformer, autoencoder

## Abstract

Recent studies have demonstrated that high-quality annotated data are crucial for segmentation performance. However, incomplete or corrupted mask annotations remain common, limiting supervised learning. To address this, we introduce a mask-reconstruction task, referred to as masked segmentation label modeling (MSLM), which refines partially occluded labels by leveraging visible regions without manual annotations. We further propose the label masked autoencoder (L-MAE), which identifies erroneous regions and reconstructs them through contextual inference. The L-MAE fuses incomplete labels and corresponding images into a unified map for reconstruction, and an image patch supplement (IPS) algorithm restores missing image information, improving the average mean intersection over union (mIoU) by 4.1%. To validate the L-MAE, we train segmentation models on a degraded and L-MAE-enhanced Pascal VOC dataset, with the latter achieving a 13.5% mIoU improvement. The L-MAE attains predict area (PA)-mIoU scores of 91.0% on Pascal VOC 2012 and 86.4% on Cityscapes, outperforming state-of-the-art supervised segmentation models.

## Highlights

• Label masked autoencoder enhances incomplete mask labels for semantic segmentation
• Multi-mask ratio inference generates labels with varying completeness for segmentation
• Integrates image-label features and restores details to improve segmentation accuracy

## The bigger picture

Semantic segmentation is a process by which a computer assigns a label to each pixel in an image, helping identify, for example, a road, pedestrian, tree, or tumor. These machine learning methods are usually trained on annotated image datasets labeled by humans. Meticulous labeling by human annotators, however, is often slow and costly, and existing annotated datasets may have errors or other flaws that limit their usefulness. Re-annotating such image datasets is often prohibitively expensive. Here, we present a method that can be used to automatically correct defective annotations. Methods such as this one could reduce the time that humans spend on relabeling tasks and help advance the development of computer vision applications, especially ones that require precise image segmentation.

## Graphical abstract

**Figure:**
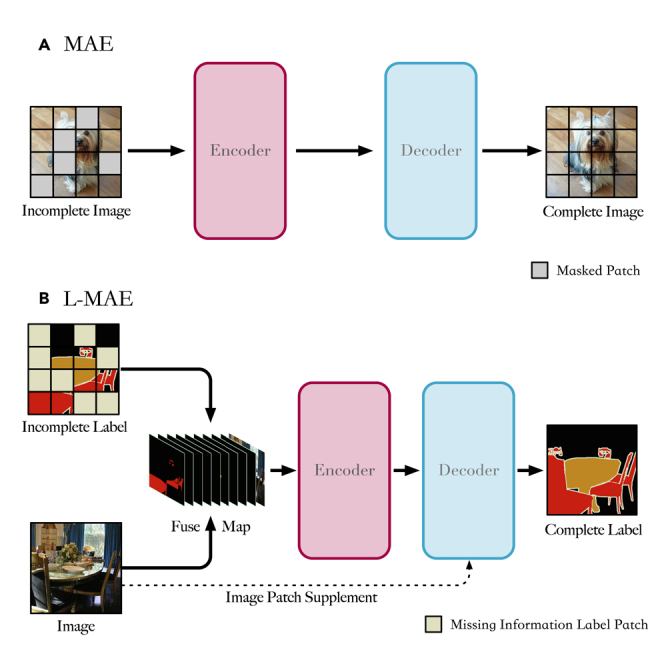

## Teaser

Many segmentation datasets carry gaps or noise in their annotations, which blunts model training. Here, the authors present a label-image fusion approach that learns to fill missing or corrupted regions. By turning imperfect labels into dependable supervision, it upgrades existing datasets and lifts accuracy without fresh hand labeling. The idea offers a simple, scalable approach to maintaining and expanding datasets across benchmarks and application domains.

## Introduction

There has been a great deal of prior work on semantic segmentation, both in deep learning research and in the context of specific applications such as medical imaging and remote sensing. These models are tailored for pixel-level semantic analysis of visual data, including images and videos, and offer technical support for diverse applications. To achieve satisfactory performance, large-scale semantic segmentation models rely on extensive datasets, and the models related to professional fields require professionals in the field to participate in creating the datasets. However, compared with other tasks, the data-labeling work of semantic segmentation models is more complex and challenging, so it is prone to labeling inaccuracies, leading to broken labels. Semi-supervised semantic segmentation methods leverage the synergistic potential between labeled and unlabeled data to enhance model generalization.^1^^,^^2^^,^^3^ This is typically achieved through the implementation of self-supervised learning mechanisms or consistency regularization strategies. A widely adopted approach involves pseudo-label generation, wherein an initial model is trained on the labeled dataset and subsequently used to generate pseudo-labels for the unlabeled data. These pseudo-labels are then combined with the original labeled data to iteratively optimize the model. However, in scenarios involving imprecise annotations, the quality of the generated pseudo-labels may degrade due to the influence of noisy labels, thereby impairing the model’s learning effectiveness and overall performance.

To solve this problem, in addition to re-labeling the data, the following methods are included in the production of large datasets or datasets in specialized fields: (1) assigning an image to multiple people for annotation and then checking the consistency,^4^^,^^5^^,^^6^ (2) conducting error analysis on existing annotations and then giving guidance to relevant workers,^7^^,^^8^ (3) using a semi-supervised semantic segmentation model for dataset amplification,^9^^,^^10^ and (4) using the iterative annotation method to first annotate a small part of the data. Then, a simple model is trained with this part of the data, and the remaining unlabeled pictures are preliminarily labeled with this model. Finally, the areas mismarked are manually revised, and so on.^1^^,^^2^^,^^3^^,^^11^ Among the above methods, manual methods can significantly increase the cost of producing datasets. In contrast, semi-supervised semantic segmentation models and iterative labeling methods may not optimally leverage labeled data with inaccuracies, so-called “broken labels,” which may result in inefficient resource utilization.^12^^,^^13^^,^^14^^,^^15^ Alternatively, we may incorporate these imprecise and precise annotated labels in the training dataset for a semi-supervised semantic segmentation model. In that case, this may lead to a decline in the model’s performance.

Our study primarily addresses the challenges posed by incomplete or inaccurate annotations in the data-labeling process, distinguishing itself from the traditional paradigm of semi-supervised learning. Conventional semi-supervised learning frameworks typically assume that datasets are composed of two distinct subsets: one with fully labeled and accurate annotations and the other entirely unlabeled. In contrast, the scenarios we address involve labeled data that may be incomplete and imprecise. These imperfect annotations pose significant challenges for direct integration into standard semi-supervised learning frameworks, as they can adversely affect model performance. To tackle this issue, our research emphasizes leveraging the latent information embedded in these incomplete annotations while preserving them. By integrating advanced data augmentation strategies and algorithmic optimization techniques, we aim to enhance the training efficiency and overall performance of models, addressing critical limitations in existing approaches and broadening their applicability to real-world datasets.

To fully use the existing broken labels, we propose a novel task—masked segmentation label modeling (MSLM). Unlike conventional approaches, MSLM performs masking and reconstruction on the fuse map, which is generated by merging image and label information. During the training phase, the proposed method incorporates image context to comprehensively extract the semantic features embedded in the labels during the reconstruction process. In the inference phase, it further refines the masked regions by leveraging both the unmasked label information and the complete image data. With an appropriate selection of masking regions, this approach enables a refined overall labeling.

Our model design is divided into a training stage and an inference stage. In the training stage, the label masked autoencoder (L-MAE) will mask and reconstruct the label. To cover the complex completion scenes in actual situations, we use a mixture of three strategies: random mask, background-first mask, and label-first mask for the masking strategy. Experiments have shown that the effect of mixed use of the three strategies is significantly better than that of using them alone. At the same time, to allow the model to reconstruct the covered area based on image information, we designed the stack fuse algorithm to fuse label and image data. We use label classification based on the layered design idea to highlight the label’s information after fusion.

Experiments have proven that the fusion strategy used in the model is better than other strategies. Considering the uniformity of the input size to the encoder during the masking step, the model can only mask the entire fused image and label. The circumstance will cover not only the label but also the image. When the model uses zero values to pad the data and restore the input size before passing them into the decoder, the image information within the masked area may be lost during the decoding process. This occurs because the decoder is unable to discern whether the zero values in the masked area originate from actual image content or are artificially introduced placeholders. Consequently, when zeros are used to pad the masked areas, the decoder may overly rely on the contextual information from the surrounding non-masked regions while reconstructing the image, leading to an inability to accurately recover the content of the masked sections. We introduce the image patch supplement (IPS) algorithm in this context. Before transmitting data from the encoder to the decoder, we employ the corresponding image patch to restore the information to its original size. Empirical evidence consistently demonstrates that models incorporating the IPS algorithm outperform those that do not, particularly in terms of completion performance.

**Figure 1:**
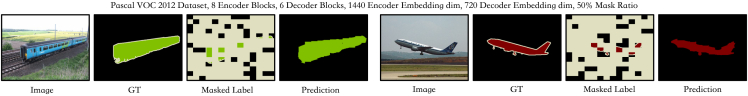
The performance of the label masked autoencoder “Masked label” denotes randomly masked complete label. As the “prediction” shows, the follow-up mask-reconstruct pipeline will complete the masked area.

Finally, to ensure fair and consistent comparison with existing methods, we propose a novel evaluation metric termed predict area mean intersection over union (PA-mIoU), which specifically measures the mIoU within regions requiring reconstruction. Given the varying degrees of label incompleteness encountered during inference, it becomes necessary to adapt the training process according to different mask ratios. It is observed that regions lacking annotations predominantly correspond to background areas. Consequently, after partitioning labels into patches, we calculate the proportion of background pixels within each patch and utilize these proportions to assign appropriate mask ratios during L-MAE training. During inference, the hybrid masking strategy adopted in training is replaced by a selective masking approach, which preferentially masks patches containing higher proportions of background pixels for subsequent reconstruction. The results are shown in Figure 1.

In summary, our contributions are 2-fold.

• We propose a mask-label enhancement method, the L-MAE, which is able to augment the label quality of datasets with incomplete mask labels to improve the performance of supervised semantic segmentation. Additionally, we design a multi-mask ratio architecture in the inference stage, which generates mask labels with varying ranges for input samples, to accommodate diverse segmentation task requirements.
• To enhance the performance of the model, we introduce two core algorithms: stack fuse and IPS. The stack fuse algorithm is designed to more effectively integrate image and label information, while the IPS algorithm aims to address the issue of supplementing image information following the fusion of maps.

The rest of this paper is organized as follows. The second section introduces recent related works. In the third section, we describe the proposed method in detail. The fourth section presents the experimental results. The final section concludes the paper.

## Related work

### Vision transformer and MAE

The vision transformer (ViT) architecture^16^ represents a seminal advancement in the application of pure transformer models to visual recognition tasks. Unlike traditional convolutional neural networks (CNNs), ViT processes input images by partitioning them into a sequence of fixed-size, regularly spaced patches. Each patch is then linearly embedded and augmented with positional encoding to retain spatial information, after which the resulting sequence is fed into a standard transformer encoder. This architecture achieves a favorable trade-off between computational efficiency and predictive accuracy, demonstrating competitive performance on image classification benchmarks. Importantly, ViT addresses a long-standing challenge in computer vision: the effective integration of positional awareness within transformer-based image representations. Empirical results further suggest that ViT scales robustly with increasing model capacity and dataset size. Nevertheless, its reliance on large volumes of labeled training data poses practical limitations in real-world applications.

To mitigate the dependence on extensive annotation, He et al. proposed the MAE framework,^17^^,^^18^ drawing inspiration from recent progress in self-supervised language modeling techniques such as bidirectional encoder representations from transformers (BERT).^19^ The MAE introduces a novel pre-training paradigm in which the model learns visual representations by reconstructing randomly masked regions of input images. The architecture consists of two distinct modules: (1) a high-capacity encoder that processes only the visible patches and (2) a lightweight decoder that reconstructs the full image by leveraging the latent representations in conjunction with mask tokens. Experimental evaluations reveal that masking a substantial portion of the image—typically around 75%—constitutes an effective pretext task for self-supervised learning. This dual-module design offers several key advantages: significantly faster convergence during training (up to 3× speedup), improved parameter efficiency, and superior performance on downstream vision tasks compared to conventional supervised methods.

In addition to random masking, recent studies have explored more advanced masking strategies, including learning-based adaptive masking and predefined multi-scale masking.^20^^,^^21^^,^^22^^,^^23^ Predefined masking strategies rely on handcrafted rules or heuristics, such as masking fixed spatial regions or selecting patches based on saliency priors, which offer simplicity and controllability in specific domains. In contrast, learning-based methods dynamically determine mask positions based on image content or attention scores, while multi-scale masking divides image patches at varying granularities to enhance semantic coverage. For instance, refinement-based masking techniques, such as the adaptive-masking-over-masking strategy proposed in Amom,^22^ dynamically update masked regions to enhance decoder refinement and improve encoder optimization. Additionally, multi-scale or learning-based masking approaches, such as BUS-M2AE (Breast UltraSound Multi-scale Masked AutoEncoder),^21^ further improve representation quality by targeting diverse semantic granularities.

### Semantic segmentation and semi-supervised semantic segmentation model

Semantic segmentation integrates image classification, object detection, and image segmentation, aiming to partition an image into distinct regional blocks, each with a specific semantic meaning, achieved through dedicated techniques. Subsequently, the semantic category of each regional block is determined, facilitating the progression of semantic reasoning from low-level to high-level information. Ultimately, the result is a segmented image with pixel-wise semantic annotations. Presently, the most widely adopted methods for image semantic segmentation rely on CNNs. Notably, these networks predominantly comprise convolutional layers with two prevalent architectural paradigms: symmetric models (e.g., fully convolutional network [FCN],^24^ SegNet,^25^ and UNet^26^) and dilated architectures (e.g., RefineNet,^27^ PSPNet,^28^ and Deeplab series^29^^,^^30^^,^^31^). Numerous outstanding semantic segmentation models have emerged in the era of the transformer’s prominence. An exemplar, SegNext,^32^ has garnered acclaim for surpassing its predecessors in semantic segmentation performance. This success can be attributed to its efficient computational design and utilization of the transformer’s encoder structure for feature extraction.

Semi-supervised semantic segmentation models extract knowledge from labeled data in a supervised way and from unlabeled data in an unsupervised manner, thus reducing the labeling effort required in the fully supervised scenario and achieving better results than in the unsupervised scenario. The commonly used methods include GAN (generative adversarial network)-like structures and adversarial training between the two networks, with one as the generator and the other as the discriminator.^33^^,^^34^^,^^35^ There are also methods for consistency regularization that include a regularization term in the loss function to minimize the difference between different predictions for the same image.^36^^,^^37^^,^^38^ There are also pseudo-labeling methods, which generally rely on predictions previously made on unlabeled data and a model trained on labeled data to obtain pseudo-labels.^39^^,^^40^^,^^41^^,^^42^^,^^43^^,^^44^ There are also methods based on contrastive learning.^45^^,^^46^ This learning paradigm groups and separates similar elements from different elements in a particular representation space.^47^^,^^48^ In contrast, our method does not rely on representation-level discrimination but rather focuses on label reconstruction through masked input modeling.

### Augmentation methods

Various conventional data enhancement methods are commonly employed to facilitate the training of highly accurate models on small, semantically split datasets. These methods typically involve basic geometric operations such as flipping, cropping, and random rotation.^49^^,^^50^ Another category of traditional transformations aims to increase the model’s training challenge by altering pixel values, including brightness, contrast, or color balance adjustments. In addition to these conventional transformations, alternative approaches involve applying different types of filters for data enhancement.^51^^,^^52^ Examples include the Sobel filter or the Canny filter for edge detection, which enhances the visibility of object edges. High-contrast vertical or horizontal edge filters can sharpen images, while Gaussian filters induce image blurring. Furthermore, adding Gaussian noise,^53^ salt-and-pepper noise, and speckle noise to images or implementing random erasure is a common data augmentation technique. Employing these methods enhances data diversity and strengthens the model’s ability to extract features for target classification. Beyond traditional augmentations, semantic-aware strategies such as ClassMix^54^ and Copy-Paste^55^ have emerged as effective techniques for combining label-consistent regions across samples. Additionally, region-level occlusion methods such CutMix^56^ or Cutout^57^ serve as data augmentation strategies by introducing structured perturbations in the input space, thereby enriching training diversity and promoting robustness, particularly under limited supervision.

As technology advances, researchers increasingly explore the application of GANs, diffusion models, and other generation networks in data enhancement for semantic segmentation datasets. Examples include AdvChain^58^ and RRVS,^59^ which generate datasets for training networks through the use of generative networks. However, traditional and novel methods based on generation networks struggle to fully utilize existing image-label information when addressing incomplete image labeling, warranting more precise enhancement effects.

## Methods

The conventional semantic segmentation model and the semi-supervised variant, which enhances the dataset, fall short of addressing potential information gaps within a single label. Our proposed L-MAE model can serve both for completion and semantic segmentation tasks.

**Figure 2:**
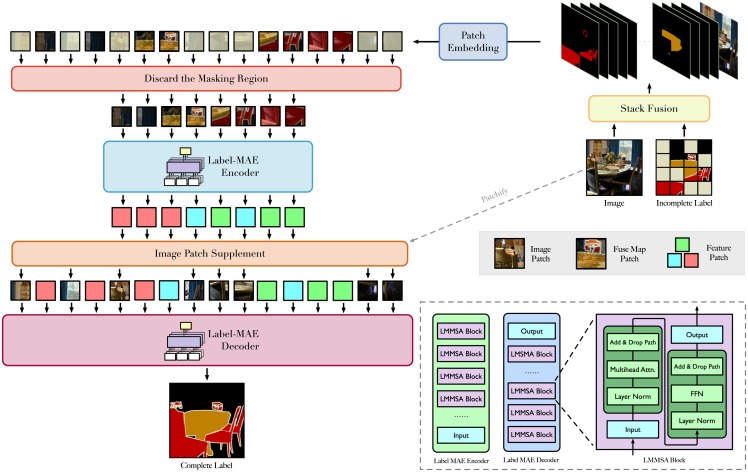
Architectural overview of the label masked autoencoder The proposed label masked autoencoder (L-MAE) framework is composed of four primary components: (1) a hierarchical fusion module for multimodal feature integration (referred to as stack fuse), (2) a context-aware encoder (L-MAE encoder), (3) a reconstruction-oriented decoder (L-MAE decoder), and (4) an information-recovery mechanism (image patch Supplement). The encoder selectively processes the visible regions of the fused feature representations, which are obtained through the integration of label and image modalities. In contrast, the decoder is designed to reconstruct the complete sequence, including masked regions, by leveraging spatial-temporal attention mechanisms. To mitigate the degradation of contextual information caused by occlusion operations from the mask selector, the image patch supplement component reinserts selected original visual patches into the masked positions. This strategy effectively preserves visual-semantic consistency and enhances the quality of the reconstructed output. Furthermore, the framework introduces a specialized L-MAE multi-head self-attention (LMMSA) mechanism, which adaptively modulates attention weights based on the preservation status of semantic labels during feature propagation. This targeted attention adjustment facilitates more effective representation learning under partially observable conditions.

This section presents detailed descriptions of the constituent modules within the L-MAE framework. To enable effective label completion, the L-MAE architecture incorporates three core innovations: (1) a hierarchical feature fusion mechanism (stack fuse), (2) a context-aware image restoration module (IPS), and (3) an adaptive inference protocol designed for scenarios involving incomplete or partially annotated data. The model architecture diagram is shown in Figure 2. The details will be illustrated in the following subsections.

### MSLM

We introduce a novel task, MSLM. Unlike conventional masked image modeling approaches that extract semantic information by masking and reconstructing images, MSLM focuses on the fuse map derived from the integration of image and label data. During training, the proposed method leverages image context to effectively extract and enhance the semantic features embedded within the label through a reconstruction process. In the inference stage, it further refines the label by performing additional reasoning on the masked regions. Provided that an appropriate masking strategy is employed to target regions prone to annotation errors and that the semantic information from the unmasked regions is fully exploited, MSLM is capable of achieving a refined and precise correction of the overall label.

### Overall architecture

Images and labels are inherently complementary in semantic segmentation datasets. Accordingly, we input both into the L-MAE so that, during reconstruction, the model can reference the original image while generating labels. The first module, stack fuse, fuses the available label cues with image features to produce a fuse map. Subsequently, the mask selector determines the set of patches to be reconstructed according to a predefined mask strategy with a specified mask ratio. After patchification and serialization, we remove the tokens corresponding to these patches and feed the remaining tokens into the L-MAE encoder. Because token removal also discards the associated image information, we introduce an IPS module that restores the fuse map content at the reconstructed locations using the original image features. Finally, the L-MAE decoder consumes the L-MAE encoder features together with the IPS augmented context to produce refined labels. The following sections describe each module in the order of the data flow.

### Stack fuse

To effectively incorporate label guidance into visual feature learning, we explored multiple fusion strategies. Initial approaches, such as direct concatenation of label maps with RGB images, resulted in limited gains due to semantic dilution and feature misalignment. To address this, we propose class-aware label embedding, which projects label maps into a class-specific feature space before fusion. This alignment facilitates more discriminative representation learning and adaptive attention allocation, leading to consistent improvements in segmentation performance with minimal computational overhead.

In this study’s implementation, the single-channel label $L\in R^{H\times W\times 1}$ is first divided according to all classes present in the dataset. Let the dataset contain *N* classes denoted by *c*_*i*_ (*i* = 1, 2, …, *N*). For each class *c*_*i*_, we locate all pixels belonging to that class in the original label and map them onto a new blank label image with the same shape as the original label. Consequently, an individual class-specific label image *l*_*i*_ is obtained for each class. These class-specific label images are then concatenated with the original image, resulting in a fused map $F\in R^{H\times W\times \left(N+3\right)}$. The process can be formulated as follows:

(Equation 1) $$F=\text{Concat}\left(l_{1},l_{2},\ldots ,l_{N},image\right)\text{.}$$

Prior to subsequent processing stages, the fused feature map undergoes patch embedding.^60^ Given a predefined patch dimension *p*, the input feature tensor $F_{f}\in R^{L\times \left(N+3\right)}$ is initially derived through spatial discretization, where $L=\frac{H\times W}{p^{2}}$ quantifies the total patch count. This embedded representation is subsequently projected into a latent semantic space via learnable linear transformation,^61^ yielding dimensionally reduced features $F_{f}\in R^{L\times e^{\prime }}$, where *e*′ denotes the encoder’s embedding dimension. The positional encoding mechanism is formally expressed as

(Equation 2) $$PE\left(pos,2i\right)=F_{f}^{i}+\sin\left(pos/10000^{2i/e^{\prime }}\right)\;i=1,2,\ldots ,e/2\;\text{and}$$

(Equation 3) $$PE\left(pos,2i+1\right)=F_{f}^{i}+\cos\left(pos/10000^{2i/e^{\prime }}\right)\;i=1,2,\ldots ,e/2\text{,}$$

where *i* indexes the encoder embedding and *pos* indexes each patch.

### Mask strategy

To enable the model to learn completion methods in various application scenarios, we design three different rules for masking labels during training, namely, random mask, background-first mask, and label-first mask.

When performing the masking operation, this study divides the image into multiple tokens based on the specified *patch*_*size*. Each token is subsequently indexed from 0 to *patch*_*size*^2^ in a top-down, left-to-right sequence. The random strategy shuffles these token indices and selects the first *patch*_*size*^2^ × *mask*_*ratio* tokens for masking, thereby covering a broad spectrum of potential cases, which is conceptually consistent with the PatchDropout,^62^ although the design motivation differs. PatchDropout operates only on images, whereas our random masking is applied to fused image-label blocks to preserve image-label correspondence during reconstruction. However, in instances where the majority of missing label information clusters around a single object, the random strategy may necessitate additional training epochs. To address this issue, the label-first strategy targets regions bearing a high proportion of object labels. Specifically, it sorts tokens in descending order based on the quantity of background-classified pixels, selects the top *patch*_*size*^2^ × *mask*_*ratio* tokens for masking, and subsequently reconstructs them via the model. This approach ensures robust completion even when most of an object’s label data are absent. Moreover, during preliminary training and validation, the model was observed to erroneously interpret the original background areas as proximate objects, prompting the design of the background-first strategy. This latter method prioritizes discarding tokens with a high background proportion by reversing the sorting order (i.e., ascending), thereby reinforcing the model’s capacity to accurately represent background regions.

In the actual training process, in order to enable the model to complete an object that is missing most of the annotation information, to correctly predict the background part, and to generalize to other common situations, we mixed the random mask strategy, label-first mask strategy, and background-first mask strategy at a ratio of 1:2:2. Experiments show that when using a mixed strategy, the completion effect of labels is significantly better than the completion effect of using each of the above strategies alone.

### L-MAE encoder and decoder

The proposed L-MAE model adopts an asymmetric encoder-decoder architecture, where the encoder operates exclusively on the visible patches, while the decoder processes features corresponding to all patches, including both visible and masked regions.

#### IPS

**Figure 3:**
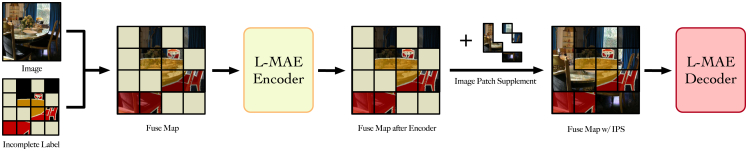
The overview of the image patch supplement process Before the fuse map is sent to the decoder, we use the image’s corresponding patch information to complete the size of the fuse map instead of using 0. The algorithm can avoid the loss of the image information at the corresponding position due to masking the fuse map by patch.

The L-MAE uses the method of masking and reconstruction to train and learn how to complete an incompletely labeled label. To ensure the uniformity of size, the masking algorithm will be directly based on the fuse map. Since both image and label information are recorded in the fuse map, the direct covering will cover up the label and image information simultaneously, which results in a decrease in prediction accuracy due to missing image information in subsequent completion operations. The workflow is shown in Figure 3.

Unlike the MAE, which employs zero or normally distributed padding,^63^ we propose a dedicated algorithm termed IPS to mitigate information loss in masked regions. Specifically, each image patch is first embedded via the patch-embedding operation, mapping it to a vector $F_{di}\in R^{L\times d^{\prime }}$, where *d*′ denotes the decoder embedding dimension. Based on the indices of the previously discarded patches, the corresponding vectors are retrieved from *F*_*di*_ and subsequently inserted into the encoder output $F_{e}\in R^{\left(L-l\right)\times d^{\prime }}$, reconstructing a full sequence for the decoder. This process has been empirically shown to substantially improve the mIoU within the prediction area.

#### Encoder and decoder

The L-MAE framework adopts LMMSA (L-MAE multi-head self-attention) blocks as the fundamental building unit. The encoder comprises *N* LMMSA blocks (default: *N* = 12), while the decoder consists of *M* LMMSA blocks (default: *M* = 8). Given an *input* to either the encoder or decoder, each LMMSA block is formulated as

(Equation 4) x=input+DropPath(MSA(LayerNorm(input)))and

(Equation 5) output=x+DropPath(FFN(LayerNorm(x))),

where FFN denotes a feedforward neural network.

The multi-head attention module (*MulAttn*) performs multiple self-attention operations in parallel, each corresponding to a distinct attention head. The outputs from all heads are concatenated and linearly projected to yield the final representation. Each head is defined by learnable weight matrices *W*^*Q*^, *W*^*K*^, and *W*^*V*^, which project the input *X* into query, key, and value matrices:

(Equation 6) $$Q_{i}=W_{i}^{Q}X,K_{i}=W_{i}^{K}X,{\text{and}\;V}_{i}=W_{i}^{V}X\text{.}$$

The attention mechanism computes the similarity between *Q*_*i*_ and *K*_*i*_ via scaled dot-product attention, followed by softmax normalization, and applies the resulting weights to *V*_*i*_:

(Equation 7) $$Z_{i}=\text{softmax}\left(\frac{Q_{i}K_{i}^{T}}{\sqrt{d_{k}}}\right)V_{i},\text{and}$$

(Equation 8) $$MSA\left(Q,K,V\right)=\text{Concat}\left(Z_{1},\ldots ,Z_{h}\right)W^{O}\text{.}$$

Here, *h* denotes the number of attention heads, and $W^{O}\in R^{hd_{v}\times d_{\text{model}}}$ is the output projection matrix. The projection matrices satisfy $W^{Q},W^{K}\in R^{d_{\text{model}}\times d_{k}}$, and $W_{i}^{V}\in R^{d_{\text{model}}\times d_{v}}$. Each attention head outputs a vector *Z*_*i*_. In practice, as adopted in the original ViT, we set *h* = 8 and use *d*_*k*_ = *d*_*v*_ = *d*_model_/*h* = 64. Despite using multiple heads, the computational complexity remains comparable to single-head attention**.** Each LMMSA block also includes a feedforward network (FFN), which applies a two-layer MLP (multi-layer perceptron) with rectified linear unit (ReLU) activation, followed by layer normalization^64^:

(Equation 9) $$\text{FFN}\left(x\right)=\text{ReLU}\left(W_{1}x+b_{1}\right)W_{2}+b_{2}\text{.}$$

While the same transformation is applied across spatial positions, the parameters of FFNs are unique to each layer in the network.

### Inference phase

**Figure 4:**
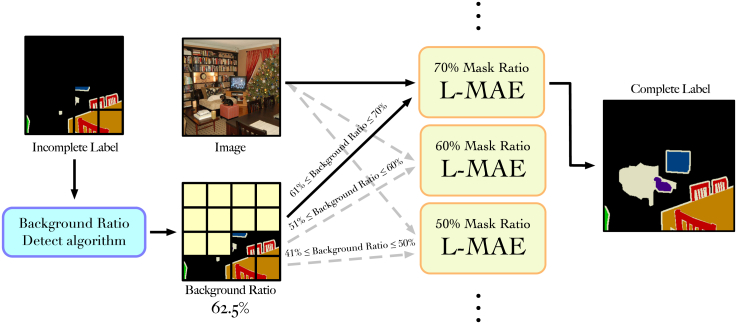
Inference phase of label masked autoencoder When an image-label pair is input, the proportion of the background part of the label will be calculated first, and according to the calculation results, the image-label pair will be sent to the L-MAE with the corresponding mask ratio.

In the inference phase, as shown in Figure 4, we train various mask ratio L-MAE models. When an image-label pair is input, the label in it will be detected, and based on the proportion of its background part, it will be input to the enhancement, which is performed in the L-MAE of the mask ratio that conforms to this ratio. During the enhancement process, the background-first algorithm will be used to mask the blocks with a high background proportion. After the features pass through the IPS module and the decoder, the model can complete the initially missing parts.

### Loss design

For the predicted label distribution *x* generated by the pipeline and the corresponding ground-truth label *y*, we adopt a cross-entropy loss function^65^ to optimize the alignment between the two modalities. Specifically, the objective encourages similarity between *x* and its matched label *y* while penalizing associations with unrelated classes. To address class imbalance in semantic segmentation, we introduce a dynamic weighting scheme that adjusts the contribution of each class based on its relative frequency in the dataset. The complete loss computation is formulated as follows:

(Equation 10) $$w_{i}=1-1/\left(1+\beta \cdot \exp\;{\left(-\frac{x_{i}-E\left(x\right)}{V\left(x\right)+\xi }\right)}^{\gamma }\right)\text{,}$$

(Equation 11) $$l_{n}=-w_{i}\cdot \log\frac{\exp\left(x_{n,y_{n}}\right)}{\sum_{i=1}^{C}\;\exp\left(x_{n,i}\right)}\cdot 1\left\{y_{n}\neq {\hat{y}}_{n}\right\},\text{and}$$

(Equation 12) $$l\left(x,y\right)=\sum_{i=1}^{N+1}l_{i}\cdot \frac{1}{\sum_{n=1}^{N+1}w_{y_{n}}\cdot 1\left\{y_{n}\neq {\hat{y}}_{n}\right\}}\text{.}$$

Here, i∈N denotes the class index, and ${\hat{y}}_{n}$ represents the ignore index, typically corresponding to the background class, which is excluded from the loss calculation. The variables *β* and *γ* are learnable hyperparameters controlling the weight scaling behavior, while *ξ* is a small constant to ensure numerical stability. N denotes the number of semantic classes, such that the output dimension becomes N+1. The terms *x*_*n*_ and *y*_*n*_ refer to the *n*-th component of the prediction and ground-truth label, respectively. The weight $w_{y_{n}}$ corresponds to the importance assigned to class *y*_*n*_ in the loss aggregation.

### Complexity analysis

In this section, we analyze the computational complexity of four principal components within the proposed L-MAE framework: stack fuse, mask strategy, encoder, and decoder.

#### Stack fuse

A single-class label *l*_*i*_ is extracted for each class *c*_*i*_(*i* = 1, 2, …, *C*). Since the original label of size *H* × *W* must be traversed once per class, the time complexity is given by

(Equation 13) $$\Omega_{Stack\_Fuse}=HW\times C\text{,}$$

where *H* and *W* denote the label’s height and width, respectively.

#### Mask strategy

Computing the background-pixel ratio for each token requires *O*(*HW*) operations. Subsequently, sorting *N* tokens incurs an *O*(*NlogN*) cost. Therefore, the overall time complexity can be expressed as

(Equation 14) $$\Omega_{Mask}=HW+N\;\log\;N\text{.}$$

#### Encoder

Assuming the masking ratio is *r*, the encoder processes only the unmasked tokens, totaling (1 − *r*)*N* tokens. Considering that a single transformer consists of a multi-head attention module followed by an FFN, the overall time complexity of the encoder can be expressed as follows:

(Equation 15) $$\Omega_{Encoder}=L*(\underset{MSA}{\underset{︸}{4*\left(1-r\right)*N*d_{model}^{2}+2*{\left(1-r\right)}^{2}*N^{2}*d_{model}}}+\underset{FFN}{\underset{︸}{8*\left(1-r\right)*N*d_{model}^{2}}})\text{,}$$

where *d*_model_ is a fixed projection dimension, *N* represents the number of tokens input to the model, and *L* denotes the number of transformer layers.

#### Decoder

The decoder processes all *N* tokens, leading to a time complexity of

(Equation 16) $$\Omega_{Decoder}=L*(\underset{MSA}{\underset{︸}{4*N*d_{model}^{2}+2*N^{2}*d_{model}}}+\underset{FFN}{\underset{︸}{8*N*d_{model}^{2}}})\text{.}$$

## Results

In this section, we first present the implementation details of the proposed L-MAE framework. Subsequently, we conduct comprehensive comparative experiments on the Pascal VOC 2012 and Cityscapes datasets to evaluate the performance of L-MAE against both supervised and semi-supervised semantic segmentation models. Finally, we perform an extensive set of ablation studies to investigate the impact of key architectural components—including the number of encoder and decoder blocks (*EB*s and *DB*s, respectively), the dimensions of encoder and decoder embeddings, and the masking ratio—on the overall performance of the L-MAE.

### Experimental setup and evaluation metrics

#### Setup

The proposed L-MAE framework is implemented using the PyTorch library and optimized with the Adam optimizer,^66^ configured with a momentum of 0.9 and a weight decay of 0.0001. To facilitate stable convergence, we employ a learning rate scheduling strategy based on ReduceLROnPlateau with min mode, which reduces the learning rate by a specified factor when the validation loss fails to improve over a predefined number of patience epochs. Specifically, we set the patience to 5 epochs, the loss reduction threshold to 0.001, and the learning rate reduction factor to 0.8.

All experiments are conducted on an NVIDIA Tesla A40 GPU, and each model is trained for 400 epochs on both the Pascal VOC 2012 and Cityscapes datasets. For Pascal VOC, images and labels are randomly cropped to 448 × 448 and subsequently resized to 224 × 224. For Cityscapes, inputs are randomly cropped to 448 × 448 without further resizing. During training, we use a batch size of 24 for Pascal VOC and 48 for Cityscapes.

#### Metrics

Employing the conventional global mIoU metric alone for comparative evaluation of semantic segmentation models may introduce bias and inefficiency, particularly due to differing supervision strategies. Since our method operates on partially labeled data, while comparison models typically utilize unlabeled datasets, evaluating performance solely on global metrics is inadequate. To enable fair comparisons, we propose a novel metric named PA-mIoU, which specifically evaluates segmentation accuracy within masked (discarded) regions. In practice, patches selected for masking by the mask selector are indexed in a list $i\in R^{l}$, generating a binary mask $m\in R^{H\times W}$ indicating masked (value = 1) and unmasked (value = 0) regions. The PA-mIoU metric exclusively calculates performance in regions where *m* = 1, thereby objectively assessing the segmentation quality in occluded label areas.

### Comparative experiments

In comparative experiments, we evaluate the proposed L-MAE against several state-of-the-art supervised semantic segmentation methods. By contrasting the PA-mIoU of the L-MAE with the conventional mIoU metric reported by comparison methods, we observe that PA-mIoU consistently improves as the mask ratio decreases. Specifically, when the mask ratio reaches 50%, the L-MAE surpasses the performance of existing state-of-the-art approaches.

#### Results on Pascal VOC 2012

The Pascal VOC 2012 dataset^67^ is an expanded version of the original Pascal VOC 2007, comprising a total of 11,530 images. For semantic segmentation, the training and validation sets of VOC2012 aggregate images from the years 2007 through 2012, including 2,913 images divided into 2,513 for training and 400 for validation. In comparison with current supervised semantic segmentation models, our L-MAE achieves 94.6% global mIoU and 91.0% PA-mIoU at a mask ratio of 50% and 92.6% global mIoU and 89.1% PA-mIoU at a mask ratio of 60%. As indicated at the top of Table 1, our method significantly surpasses the existing state-of-the-art methods. Additionally, we compare our approach with prominent semi-supervised segmentation models, specifically U2PL (Using Unreliable Pseudo Labels) and S4MC (Semi-Supervised Semantic Segmentation via Marginal Contextual Information). As demonstrated in the table, L-MAE consistently outperforms these semi-supervised methods by a margin exceeding 5% mIoU under similar masking conditions, further validating the superior performance of our proposed framework.

**Table 1:** Comparison with supervised state-of-the-art semantic segmentation methods on Pascal VOC 2012 datasets

Methods	Aero	Bike	Bird	Boat	Bottle	Bus	Car	Cat	Chair	Cow	Table	Dog	Horse	Mbike	Person	Plant	Sheep	Sofa	Train	TV	mIoU
RefineNet^27^	95.0	73.2	93.5	78.1	84.8	95.6	89.8	94.1	43.7	92.0	77.2	90.8	93.4	88.6	88.1	70.1	92.9	64.3	87.7	78.8	84.2
ResNet38^68^	96.2	75.2	95.4	74.4	81.7	93.7	89.9	92.5	48.2	92.0	79.9	90.1	95.5	91.8	91.2	73.0	90.5	65.4	88.7	80.6	84.9
PSPNet^28^	95.8	72.7	95.0	78.9	84.4	94.7	92.0	95.7	43.1	91.0	80.3	91.3	96.3	92.3	90.1	71.5	94.4	66.9	88.8	82.0	85.4
Deeplabv3^31^	96.4	76.6	92.7	77.8	87.6	96.7	90.2	95.4	47.5	93.4	76.3	91.4	97.2	91.0	92.1	71.3	90.9	68.9	90.8	79.3	85.7
EncNet^69^	95.3	76.9	94.2	80.2	85.3	96.5	90.8	96.3	47.9	93.9	80.0	92.4	96.6	90.5	91.5	70.9	93.6	66.5	87.7	80.8	85.9
DFN^70^	96.4	78.6	95.5	79.1	86.4	97.1	91.4	95.0	47.7	92.9	77.2	91.0	96.7	92.2	91.7	76.5	93.1	64.4	88.3	81.2	86.2
SDN^71^	96.9	78.6	96.0	79.6	84.1	97.1	91.9	96.6	48.5	94.3	78.9	93.6	95.5	92.1	91.1	75.0	93.8	64.8	89.0	84.6	86.6
Deeplabv3+^72^	97.0	77.1	97.1	79.3	89.3	97.4	93.2	96.6	56.9	95.0	79.2	93.1	97.0	94.0	92.8	71.3	92.9	72.4	91.0	84.9	87.8
ExFuse^73^	96.8	**80.3**	97.0	82.5	87.8	96.3	92.6	96.4	53.3	94.3	78.4	94.1	94.9	91.6	92.3	81.7	94.8	70.3	90.1	83.8	87.9
MSCI^74^	96.8	76.8	97.0	80.6	89.3	97.4	93.8	97.1	56.7	94.3	78.3	93.5	97.1	94.0	92.8	72.3	92.6	73.6	90.8	85.4	88.0
MARS^75^	89.3	42.0	88.8	72.9	79.5	92.7	86.2	94.2	40.3	91.4	58.8	91.1	88.9	81.9	84.6	63.6	91.7	91.7	85.3	57.3	77.7
DHR^76^	93.3	42.6	86.6	74.8	72.3	95.0	88.3	95.1	41.6	90.9	71.2	93.3	93.3	86.8	85.7	73.9	93.9	63.4	81.8	56.8	79.8
CoSA^77^	93.3	47.0	84.2	60.2	75.0	87.7	81.7	92.0	34.5	87.8	59.6	86.2	86.3	84.9	82.8	68.2	87.4	63.9	67.7	61.6	75.2
MRFM^78^	**97.1**	78.6	**97.1**	80.6	89.7	**97.3**	**93.6**	**96.7**	59.0	**95.4**	81.1	93.2	**97.5**	**94.2**	**92.9**	72.3	93.1	74.2	91.0	85.0	88.4
**L-MAE w/m=0.6**	89.5	58.8	92.3	86.4	91.3	94.9	89.6	95.3	79.1	93.7	89.6	93.4	91.0	90.5	89.4	85.6	96.1	93.0	96.0	87.5	**89.1**
**L-MAE w/m=0.5**	89.9	64.5	91.4	**89.2**	**92.1**	95.9	90.6	96.4	**82.1**	94.6	**91.2**	**94.7**	94.3	92.4	91.1	**87.8**	**97.8**	**94.0**	**96.5**	**93.4**	**91.0**

For fair evaluation, the proposed method is assessed using the PA-mIoU metric, and other models using the mIoU metric. Here, “*m*” denotes the mask ratio, and the optimal hyper-parameter configuration selected for L-MAE is as follows: Encoder Blocks = 8, Decoder Blocks = 6, Encoder Embedding Dimension = 1440, and Decoder Embedding Dimension = 720.

#### Results on Cityscapes

The Cityscapes dataset^79^ comprises 5,000 pixel-level annotated images, covering semantic and instance labels from street scenes collected across 50 cities in Germany and neighboring countries during spring, summer, and autumn. For evaluation purposes, experiments were conducted with mask ratios set to 50% and 60%, respectively. At a 50% mask ratio, our proposed L-MAE achieves 90.5% global mIoU and 86.4% PA-mIoU, whereas at a 60% mask ratio, it attains 89.0% global mIoU and 85.6% PA-mIoU. As illustrated at the bottom of Table 2, the proposed L-MAE significantly outperforms previous state-of-the-art methods. Additionally, comparative experiments with the semi-supervised semantic segmentation method U2PL demonstrate the superiority of our model. As shown in Table 3, our approach exhibits consistent advantages in mIoU performance under both comparable mask ratios (87.5% versus 80% and 75% versus 70%) and identical masking conditions (50%).

**Table 2:** Comparison with supervised state-of-the-art semantic segmentation methods on Cityscapes datasets

Methods	Road	Swalk	Build	Wall	Fence	Pole	Tlight	Tsign	Veg.	Terrain	Sky	Person	Rider	Car	Truck	Bus	Train	Mcycle	Bicycle	mIoU
VPLR^80^	98.8	87.8	94.2	64.1	65.0	72.4	79.0	82.8	94.2	74.0	96.1	88.2	75.4	96.5	78.8	94.0	91.6	73.7	79.0	83.5
HRNet_OCR^81^	98.8	88.3	94.1	66.9	66.7	73.3	80.2	83.0	94.2	74.1	96.0	88.5	75.8	96.5	78.5	91.8	90.1	73.4	79.3	83.7
P_Deeplab^82^	98.8	88.1	94.5	68.1	68.1	74.5	80.5	83.5	94.2	74.4	96.1	89.2	77.1	96.5	78.9	91.8	89.1	76.4	79.3	84.2
iFLYTEK-CV	98.8	88.4	94.4	68.9	66.8	73.0	79.7	83.3	94.3	74.3	96.0	88.8	76.3	96.6	84.0	**94.3**	91.7	74.7	79.3	84.4
SegFix^83^	98.8	88.3	94.3	67.9	67.8	73.5	80.6	83.9	94.3	74.4	96.0	89.2	75.8	**96.8**	83.6	94.1	91.2	74.0	80.0	84.5
HMSA^84^	**99.0**	89.2	94.9	71.6	69.1	75.8	**82.0**	**85.2**	94.5	75.0	**96.3**	**90.0**	79.4	96.9	79.8	94.0	85.8	77.4	81.4	85.1
**L-MAE w/m=0.6**	97.4	89.1	94.1	89.2	90.7	74.8	66.0	71.7	93.9	88.6	94.5	81.7	73.8	93.6	89.6	89.8	90.3	79.3	78.0	**85.6**
**L-MAE w/m=0.5**	98.0	**91.9**	**95.8**	**91.9**	**93.0**	**81.4**	74.4	78.6	**95.7**	**91.1**	95.8	86.2	**79.7**	95.3	**91.9**	91.7	**92.1**	**83.6**	**82.9**	**86.4**

Consistent with the evaluation on Pascal VOC 2012, the proposed method is assessed using the PA-mIoU metric, and other models using the mIoU metric, where “*m*” denotes the mask ratio. We also use the following abbreviations for class labels: swalk (sidewalk), tsign (traffic sign), tlight (traffic light), Veg. (vegetation), and mcycle (motorcycle).

**Table 3:** Comparison with semi-supervised semantic segmentation state-of-the-art U2PL on Pascal VOC 2012

Method	Mask ratio	mIoU / PA-mIoU
U2PL	87.5%	79.01
	75%	79.30
	50%	80.5
S4MC	87.5%	79.67
	75%	79.85
	50%	81.1
L-MAE(w/IPS) (Measured with PA-mIoU)	80.0%	85.5
	70%	90.1
	50%	91.3

The metric mIoU for the L-MAE is PA-mIoU for fairly.

### Label augmentation experiment

**Figure 5:**
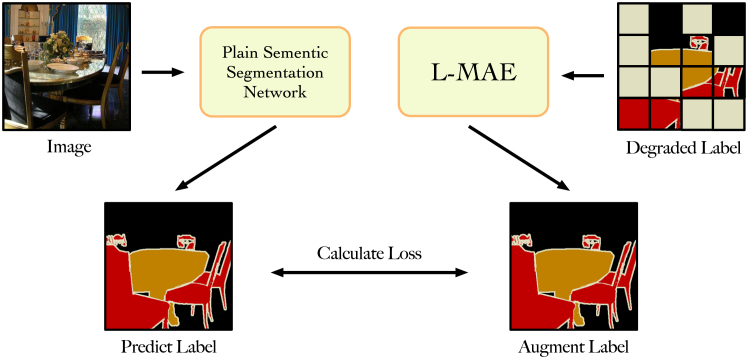
Label augmentation experiment study pipeline The plain semantic segmentation network will calculate the loss with the L-MAE-regenerated label.

We conducted the label augmentation experiment to assess whether enhancing the dataset with the L-MAE yields performance improvements in conventional semantic segmentation models, as illustrated in Figure 5. Within this experiment, we intentionally degraded the Pascal VOC dataset by randomly obscuring 50% of the data. We then employed this degraded dataset to train FCN and UNet models for 300 iterations. Subsequently, we applied the L-MAE to enhance the degraded dataset and employed this improved dataset to retrain the FCN and UNet models for another 300 iterations. As shown in Table 4, the results demonstrate notable enhancements in the performance of the trained FCN and UNet models on the test set, with improvements of 13.4% and 11.7%, respectively, compared to the original dataset. These findings strongly affirm the effectiveness of the L-MAE in practical scenarios.

**Table 4:** Compare the performance difference between ordinary semantic segmentation networks trained using the L-MAE enhancement method and unenhanced ones

Network	mIoU w/L-MAE	mIoU w/o L-MAE
FCN	43.5%	57.9%
UNet	59.5%	71.2%

### Ablation study

To assess the contribution and performance impact of individual components within our proposed model, we conduct a detailed ablation study in this section. Experiments are performed using the Pascal VOC dataset with an input size of 224 × 224.

#### Parameter setting analysis

As illustrated in Table 5, we investigate the performance of the proposed L-MAE under various hyperparameter configurations. Specifically, four key hyperparameters are analyzed: the number of encoder blocks (*EB*s), the number of decoder blocks (*DB*s), the encoder embedding dimension (*ED*), and the decoder embedding dimension (*DD*). Results indicate that performance generally declines as embedding dimensions decrease, given a fixed number of *EB*s and *DB*s. Notably, an exception occurs in the case of *EB* = 12 and *DB* = 8, where setting *ED* = 1,440 and *DD* = 720 results in a lower PA-mIoU compared to *ED* = 1,024 and *DD* = 512, although the global mIoU remains unchanged. This observation suggests improved accuracy within the unmasked regions. Based on these experimental outcomes, we select the configuration of *EB* = 8, *DB* = 6, *ED* = 1,440, and *DD* = 720 for subsequent analyses.

**Table 5:** Comparisons across different hyperparameter configurations

*EB*	*DB*	*ED*	*DD*	#Parameters (M)	FLOPs (G)	mIoU	PA-mIoU
12	8	1,024	512	186 M	21 G	94.1	90.5
		1,440	720	362 M	42 G	94.1	90.4↓
8	6	1,024	512	129 M	15 G	94.1	90.3
		1,440	720	250 M	29 G	94.6↑	91.3↑
6	4	1,024	512	98 M	11 G	94.2	90.4
		1,440	720	188 M	22 G	94.6↑	91.2↑

We report the corresponding model parameters (in M) and computational complexity in terms of FLOPs (in G). All reported mIoU and PA-mIoU metrics include the background class. *EB*, the number of encoder blocks; *DB*, decoder blocks; *ED*, encoder embedding dimension; *DD*, decoder embedding dimension; M, millions; FLOPs, floating-point operations; G, billions.

#### Masking strategy

To validate the effectiveness of our masking strategies, we evaluated four settings: random masking, label-first masking, background-first masking, and a mixed scheme that combines them in a 1:2:2 ratio. The results are summarized in Table 6. We report both mIoU and PA-mIoU. The results show that the mixed scheme performs the best, improving over random masking by 0.9% on Pascal VOC and 1.3% on Cityscapes, indicating a clear advantage. In contrast, background-first masking alone yields the weakest performance, likely because masking only background regions provides insufficient semantic perturbation on labeled objects, limiting the model’s ability to learn robust label-reconstruction behavior. We also compared our method with the masking strategy used in BUS-M2AE for medical imaging. As shown in Table 7, under matched datasets and training schedules, the mixed strategy in the L-MAE exceeds the TMM (token-level multi-scale masking) + FMM (feature-level multi-scale masking) combination in BUS-M2AE by 1.2% and 1.7% mIoU and by 0.9% and 1.4% PA-mIoU on Pascal VOC and Cityscapes, respectively. We attribute these gains to combining random masking, label-first masking, and background-first masking in a 1:2:2 ratio, which mitigates background-biased predictions while placing greater emphasis on labeled regions. Compared with BUS-M2AE’s image-centric multi-scale masking, our approach offers better efficiency and stronger task-specific adaptability.

**Table 6:** Ablation studies on the Pascal VOC 2012 and Cityscapes datasets using three mask strategies: Random mask, background-first mask, and label-first mask

Dataset	Mask ratio	Mask strategy	mIoU	PA-mIoU	ΔmIoU	ΔPA-mIoU
Pascal VOC 2012	50%	random mask	93.7	90.2	0.0	0.0
		label first	94.3	90.6	+0.6	+0.4
		background first	87.8	82.1	−5.9	−8.1
		**mixed (1:2:2)**	**94.6**	**91.0**	**+0.9**	**+0.8**
Cityscapes	50%	random mask	89.2	84.9	0.0	0.0
		label first	90.1	85.7	+0.9	+0.8
		background first	86.9	81.3	−2.3	−3.6
		**mixed (1:2:2)**	**90.5**	**86.4**	**+1.3**	**+1.5**

We report mIoU, PA-mIoU, and the performance gains ΔmIoU and ΔPA-mIoU over the random mask baseline with the mask ratio fixed at 50%. Bolding represents the method we ultimately adopted.

**Table 7:** Comparison of masking methods on Pascal VOC 2012 and Cityscapes datasets under a fixed mask ratio of 50%

Dataset	Method	mIoU	PA-mIoU	ΔmIoU	ΔPA-mIoU
Pascal VOC 2012	**L-MAE: mixed (1:2:2)**	**94.6**	**91.0**	**+1.2**	**+0.9**
	BUS-M2AE (TMM only)	93.0	89.5	−0.4	−0.6
	BUS-M2AE (FMM only)	92.9	89.4	−0.5	−0.3
	BUS-M2AE (TMM+FMM)	93.4	90.1	0.0	0.0
Cityscapes	**L-MAE: mixed (1:2:2)**	**90.5**	**86.4**	**+1.7**	**+1.4**
	BUS-M2AE (TMM only)	88.4	84.3	−0.4	−0.7
	BUS-M2AE (FMM only)	88.3	84.2	−0.5	−0.8
	BUS-M2AE (TMM+FMM)	88.8	85.0	0.0	0.0

ΔmIoU and ΔPA-mIoU denote the performance gains compared to BUS-M2AE (TMM+FMM) on the same dataset. Bold numbers indicate the best performance for each dataset.

#### Mask ratio and IPS

**Figure 6:**
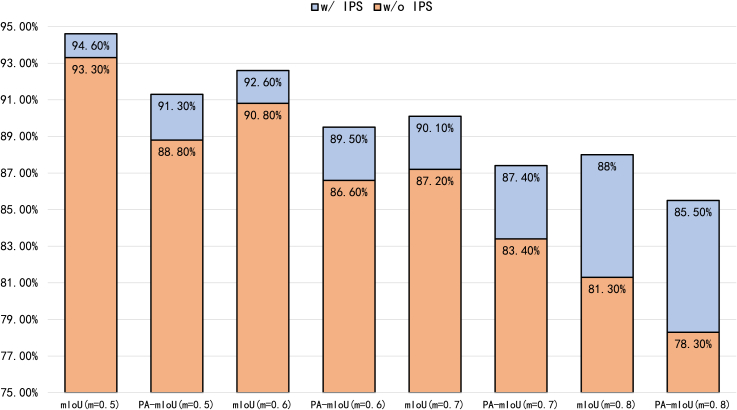
Ablation study evaluating the impact of varying mask ratios and the presence or absence of the IPS algorithm All reported mIoU and PA-mIoU metrics include the background class. Here, “*m*” denotes the mask ratio.

We investigate the effectiveness of the proposed IPS algorithm and the influence of different mask ratios. As depicted in Figure 6, after applying the IPS algorithm, the average mIoU decreases by 3.0%, whereas the average PA-mIoU declines by 4.1%. Notably, the impact of IPS varies with the mask ratio: a higher mask ratio results in a more pronounced improvement in PA-mIoU. Additionally, IPS exhibits distinct influences on mIoU and PA-mIoU; for instance, at a mask ratio of 0.7, the reduction in mIoU is smaller than that in PA-mIoU. These results indicate that IPS significantly enhances segmentation accuracy within masked regions, effectively supplementing the missing visual information at appropriate model positions. We further compare the training efficiency across different mask ratios, measured in time per batch (TPB), which denotes the training time required for each batch. As presented in Table 8, the results demonstrate that as the mask ratio increases, the TPB gradually decreases, primarily due to the reduced computational load in the encoder stage.

**Table 8:** Training time per batch (s/batch) for L-MAE at different mask ratios on the Pascal VOC datasets, using 224 × 224 inputs

Dataset	Mask ratio	TPB
Pascal VOC 2012	30%	3.694
	50%	3.328
	60%	3.124
	70%	2.917

The batch size is 24. Measurements were taken on a single NVIDIA A40 GPU. TPB, training time per batch.

#### Stack fuse

As shown in Table 9, we compared the performance impact of three different image-label fusion methods on the model. Under the premise that the parameters are set to *EB* = 8, *DB* = 6, *ED* = 1,440, and DD = 720 and the patch training strategy is randomly discarded. The fusion method that is directly concatenating the label to the image, which is called directly concat, has 72.4% PA-mIoU and 74.8% mIoU. The fusion method, in which the label is replicated and inserted into each of the three RGB channels of the image, which is called insert concat, has 75.6% PA-mIoU and 77.9% mIoU. In comparison, the method used in this article to layer labels by category, which is called layer concat, can achieve 94.6% PA-mIoU and 91.3% mIoU, fully proving the advantages of fusion strategies.

**Table 9:** Comparison with several different stack fuse methods

Method	mIoU	PA-mIoU
Directly concat	72.4%	74.8%
Insert concat	75.6%	77.9%
Directly concat	94.6%	91.3%

Directly concat concatenates the label map directly with the RGB image; insert concat involves replicating the label map and appending it to each of the three RGB channels individually; layer concat separates the label map into category-specific channels before concatenation.

### Qualitative study

#### Visualization

**Figure 7:**
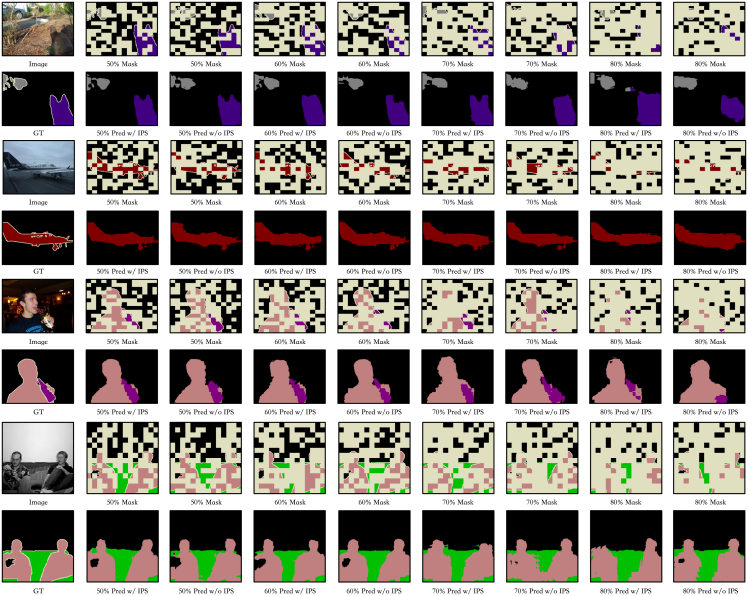
Qualitative examples with different settings We demonstrate the label completion performance at masking rates of 50%, 60%, 70%, and 80%, both with and without the use of the IPS algorithm. Additionally, we present the original images alongside the corresponding masked labels for each configuration, where the pale yellow regions indicate the areas that have been masked.

Visualization results under different experimental settings are presented in Figure 7 to illustrate the effectiveness of each component within our proposed approach. First, compared to the L-MAE without the IPS, the version employing the IPS achieves notably better performance across various mask ratios. This discrepancy arises because masking operations remove both labels and corresponding image regions, hindering the effective use of local image information in subsequent stages. Second, we observe that model performance remains relatively robust as the mask ratio increases, highlighting the stability of our framework. Finally, our model consistently produces high-quality segmentation masks, validating the overall effectiveness of the proposed L-MAE method.

#### Failure cases

**Figure 8:**
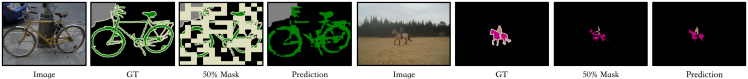
Qualitative examples of failure cases

Figure 8 presents several representative failure cases that provide valuable insights into the limitations of the proposed method. One notable type of failure occurs when reconstructing objects characterized by elongated or tubular structures. As illustrated in the left example of Figure 8, the predicted “green” region inadequately represents the complete structure of the bicycle. Another failure scenario arises from ambiguity between masked regions and complex background information, leading to confusion in segmentation. Additionally, our analysis indicates that the reconstruction accuracy of the L-MAE is reduced for small target objects due to excessive masking of fine-grained details. Although reducing the grid size could alleviate this issue, it would concurrently increase the model’s parameter count and computational overhead.

## Discussion

In this paper, we have explored the potential of leveraging MAE models for pixel-level label completion. We propose an end-to-end framework, termed the L-MAE, to effectively transfer the mask-and-reconstruct capabilities of the MAE to semantic segmentation tasks. Compared with conventional methods, the proposed L-MAE inherits the MAE’s robust pixel reconstruction ability, allowing for effective reconstruction of unknown pixel labels based on partially available annotations. The proposed IPS algorithm further enriches image features at masked regions, thereby preserving information integrity during the completion process. Additionally, the designed fusion training approach generalizes well across diverse completion scenarios, enabling the L-MAE to effectively restore missing labels under various conditions. Extensive comparative experiments and comprehensive ablation studies conducted on two widely used segmentation datasets demonstrate the effectiveness of each proposed component, verifying that our L-MAE substantially outperforms existing methods without relying on pre-trained weights.

In our analysis of the L-MAE, we have also identified some of its limitations. For instance, the model sometimes results in rough edges or excessive annotations when processing smaller objects. Moreover, attempts to generate markings for completely unannotated objects occasionally lead to significant errors. These issues may stem from the fact that, while the transformer’s attention mechanism excels at extracting global features, it overlooks smaller, localized areas, leading to deviations in handling details. To address this issue, we plan to develop a new structure focusing on global features and accurately capturing details in smaller regions. At the same time, since the L-MAE uses the transformer structure, its global context modeling capability is also valuable in 3D tasks. Next, we consider adapting the L-MAE to point-cloud or voxel-level semantic segmentation tasks, capturing long-range spatial dependencies through the transformer, and improving the understanding of complex 3D scenes.

## Resource availability

### Lead contact

Requests for further information and resources should be directed to and will be fulfilled by the lead contact, Mingzhe Liu (liumz@wzut.edu.cn).

### Data and code availability

• The datasets used in this study are publicly available. Pascal VOC^67^ and Cityscapes^79^ were used for training and evaluation of our models and for quantitative comparisons. Both datasets can be accessed for research use under their respective licenses, as described in the original publications and project pages, and are cited in the references section.
• All code for the L-MAE, along with scripts for data preprocessing, training, and evaluation, is available at GitHub and has been archived at Zenodo,^85^ which is also cited in the references.
• Any additional information required to reanalyze the data reported in this paper is available from the lead contact upon request.

## Acknowledgments

The computational experiments in this work were performed using self-funded NVIDIA A40 GPUs. We thank NVIDIA Corporation for developing this high-performance hardware platform that enabled efficient training of our mask autoencoder models. An early version of this manuscript was deposited on the arXiv preprint server under the identifier arXiv:2211.11242, which facilitated valuable feedback from the computer vision research community prior to formal peer review.

## Author contributions

J.J. and X.C. conceived of the presented idea. J.J. developed the theory and performed the computations. J.J. wrote the manuscript with support from L.Z. and X.C. All of the authors contributed to the final version of the manuscript.

## Declaration of interests

The authors declare no competing interests.

## Declaration of generative AI and AI-assisted technologies in the writing process

During the preparation of this work, the authors used ChatGPT in order to polish the writing. After using this tool or service, the authors reviewed and edited the content as needed and take full responsibility for the content of the publication.
